# Supplementary material for: Health system opportunities and challenges for PrEP implementation in Kenya: A qualitative framework analysis
Source: PLoS One. 2022 Oct 7;17(10):e0259738. doi: 10.1371/journal.pone.0259738 (PMC9543691; doi:10.1371/journal.pone.0259738)
Supplement: S1 File — (DOCX) [file pone.0259738.s001.docx]

**Appendix A: Healthcare Provider FGD Guide**

***Providers: Formative Project Phase (early 2018)***

1. **Perceptions about HIV prevention**
   1. What are the current perceptions towards HIV risk within your community? What explains these observations? Which community groups are being perceived to have high risk of new HIV infections?
   2. Which methods of HIV prevention are currently most popular in your community? *Probe ABC, PEP, PrEP, TasP.* How have these changed after the introduction of Oral PrEP?
2. **Knowledge and attitudes about KPs**
   1. What is the current opinion towards key populations (FSW, MSM/MSW, transgender) in your community? *What explains these opinions? How do these opinions shape the way the community interacts with these populations? What suggestions do you make can improve the opinions of the community towards KPs?*
   2. What is your current opinion regarding Oral PrEP as a method of preventing new HIV infections in your community? How about provision of oral PrEP to KPs, AGYW?
3. **Acceptability of PrEP**
   1. What if the current level of acceptability of PrEP as part of the HIV prevention methods in the community? Why? /Why Not? What things are making your community to embrace oral PrEP? *What is discouraging your community from embracing Oral PrEP?*
   2. What are your views about prescribing oral PrEP to adolescent girls, young women, and negative partners in sero-discordant couples?
   3. To what extend are stigma, discrimination, religious views and violence towards FSW, MSM/MSM, sexually active AGYW affecting the delivery of oral PrEP? *How can these be addressed?*
   4. What access routes are being preferred in your community to receive PrEP services*? in-reaches, outreaches, moonlights, walk-ins, peer-led approaches*
4. **Access for PrEP**
   1. Which service delivery channels are members from your community preferring to access Oral PrEP? Why? What explains differences in preferences between different users? *Probe DICEs, private facilities and public facilities*
   2. What barriers do you think willing PrEP users from your community encounter when accessing PrEP at the various health facilities? *Waiting times, HIV testing, lack of commodities, many revisits*, *high mobility, monthly refills, lack of privacy, poor attitudes among staff*
   3. What factors are promoting the use of PrEP at the facilities? *Probe client, health system, community facilitators*
   4. What suggestions can you make to the service delivery channels within your community to improve the quality of oral PrEP services?
   5. From your own opinion, how much has the introduction of PrEP affected the use of other prevention methods such as abstinence, condoms, one faithful partner? *To what extend is your community reporting more STIs, pregnancies, abortions?*
5. **Information about Oral PrEP:**
   1. What information gaps about oral PrEP have not been adequately addressed in your community? *What groups have not been reached with adequate information? What can be done to address these gaps? What avenues can this information be relayed to those who need it most?*
   2. What additional information about PrEP would you recommend to your community?

***Providers: Adapted Guide as Scale-Up Progressed***

1. **Perceptions about HIV prevention**
   1. What are the emerging issues among healthcare providers regarding the inclusion of PrEP to the existing combination prevention interventions?
   2. How are clinicians currently assessing user-risk for HIV transmission? What changes have occurred as a result of the introduction of PrEP?
   3. What tools are clinicians using as a guide in determining risk? How useful have been the NASCOP guidelines in assessing risk? How about the training toolkit?
2. **Knowledge and attitudes about KPs and HIV prevention**
   1. Which HIV prevention methods are clinicians preferring to offer? What is it that makes these methods still very popular?
   2. How you finding the interaction with KPs? What has facilitated the smooth interactions? What barriers still exist in clinician interaction with KPs? How about discussion sexual issues with KPs?
   3. What is your current opinion regarding Oral PrEP as a method of preventing new HIV infections? What explains your description?
3. **Acceptability of PrEP**
   1. How is the acceptable has PrEP been among your fellow clinicians? Why? /Why Not? What things are making your colleagues accept to prescribe oral PrEP? *What is discouraging your colleagues from prescribing Oral PrEP?*
   2. What is your view you about prescribing PrEP to KPs? *Probe about adolescent girls, young women, FSW, MSM, HIV positive peers*
   3. How much is the stigma and discrimination towards KPs affecting the delivery of PrEP? *How can that be addressed?*
4. **Access for PrEP**
   1. Where (which sections) in your health facility are clients mostly accessing oral PrEP? What informed the choice of these sections?
   2. What barriers do clients encounter when accessing oral PrEP in your health facility? What can be done address these barriers?
   3. What has facilitated the easy access of oral PrEP in your health facility by the clients? How can they be strengthened?
   4. To what extent can offering oral PrEP be affected or has been affected by other health facility activities?
   5. What barriers are you facing when delivering PrEP services in your facilities?
   6. What factors do you think can improve the quality of PrEP services?
   7. What are the trends in uptake, adherence, and revisits among the oral PrEP clients? What can be done improve these indicators? [*Probe about: use of client tracking using peer educators, use of peer clubs, peer buddies, SMS, calling, sending adherence messages, adherence counselling etc.]*
   8. How willing are you to speak to people, friends and peers about the use of oral PrEP*?* What do you think can be done to facilitate you to do that?
5. **Information about Oral PrEP:**
   1. Which are the most effective ways/methods that have been used to reach your colleagues with information about oral PrEP? Why do you say so? [Probe about: *social media, PrEP training, mentorship, peer-led approaches (on job training), print etc*.]
   2. What key most information currently being communicated to health care providers about oral PrEP? What information gaps have not been adequately addressed as far as the provision of oral PrEP is concerned?
   3. What do you think are the most important things that your colleagues would want to know about Oral PrEP? Under what circumstances would this information be provided?
   4. What additional information about oral PrEP would you like to know?

**Appendix B: Peer Educator FGD Guide**

***Peer Educators: Formative Project Phase (early 2018): English Guide***

1. **Perceptions about HIV prevention**
   1. What do you know about HIV? In what ways has HIV affected the people within your community? *Health wise? Economically?*
   2. What are the most common ways that HIV is contracted in your community? *In your opinion which groups has been MOST AFFECTED by HIV? Why do say that? What groups are LIKELY INFECTED?*
   3. When you think about the ABCs (Abstinence, be faithful and Condom) in the prevention of HIV in this community, how successful would you say has been the adherence to each of the ABCs? Why? (*probe for the most and least effective measures*)
2. **Knowledge and attitudes about methods**
   1. Which prevention method do MOST peers/community members use? What is it that makes this method the MOST popular?
   2. Have you heard of any drug that would prevent someone from getting HIV if taken appropriately? *How did you hear about it? What did you hear about it? How did you feel when you first heard about the drug? How about now?* (*probe to differentiate between PEP and PrEP*)

*I want to tell you about a new measure that is being introduced to prevent people from contracting HIV. It is known as Oral PrEP. [Read the Oral PrEP description and keep it displayed until you come to the next set of questions]. Oral PrEP is a drug that is taken to ensure that one does not contract HIV when having sexual intercourse with an infected person. If you are at risk of being exposed, you are required to take the drug seven days prior to exposure and for 28 days after possible exposure. On the other hand, if there is a possibility of you being exposed at every sexual encounter you are required to take the drug on a daily basis.*

1. **What is your opinion regarding Oral PrEP as a method of preventing HIV spread?**
2. **Acceptability of PrEP**
   1. What are the things that would make your peers/community members accept Oral PrEP? *What would discourage your peers*/community members *from taking Oral PrEP?*
   2. What groups do you feel would be most willing to accept the use of Oral PrEP? *Probe age, key populations, social groups etc*
   3. What are the things that your peers/community members would like MOST about Oral PrEP? What are the things that your peers/community members would LEAST like about Oral PrEP? Would these dislikes be enough to cause them to refuse to use the drug? Why do you say that?
   4. How would your peers/community members view someone who is taking Oral PrEP? *Probe about adolescent girls, young women, FSW, MSM, HIV positive peers*/community members, individuals in sero-discordant relationships and other individuals
   5. Do you think peers could encourage each other to get Oral PrEP? Why? Why not?
3. **Access and peer support**
   1. Where do you think your peers/community members would be most comfortable accessing Oral PrEP? Why? *Probe DICES, Public and private health facilities, outreaches*
   2. Would you be willingly participating in mobilizing and accompanying your peers/community members to access Oral PrEP? *Why? Why Not? Where would you do it from?* If you knew where Oral PrEP can be accessed would you willingly tell your peers/community members where to get it? What can motivate you to work efficiently as a peer educator/community health worker for PrEP?
   3. If you were aware that your peer/community members was sexually active, would you discuss Oral PrEP with them? Why? Why Not? *How would you do it?*
   4. What benefits would accrue from PrEP users if they were frequently meeting other PrEP users (peer support clubs)? *How do you think these meetings should be organized? How often and where? What can encourage you to participate in these meetings*
   5. Would you accept to be paired-up with a PrEP user (as peer buddy) so that you can support them? Why and why not? How do you think that should be done?
   6. Would you be willing to speak to people, friends and peers/community members about your use of PrEP*? Would you be willing to share your experiences? What do you think can be done to make you do that?*
   7. To what extend can the use of PrEP be affected by violence, fear of discrimination, fear of losing clients/partners and business? What would cause these problems? *How can PrEP be delivered to reduce these problems?*
4. **Information about Oral PrEP:**
   1. Which are the best ways/methods to reach your peers/community members with information about oral PrEP, why do you say so? *Probe for social media, peer-led approaches, print etc. What more would you like to know about Oral PrEP?*
   2. What do you think are the most important things that your peers/community members would want to know about Oral PrEP?
   3. What kind of messages about Oral PrEP could be used to make Oral PrEP acceptable to your peers/community members? Under what circumstances would you provide this information?
   4. What additional information about PrEP would you like to know?

***Peer Educators: Formative Project Phase (early 2018): Kiswahili Guide***

1. **Ufahamu na mitazamo kuhusu kuzuia HIV**
   1. Ni nini unafahamu kuhusu HIV? HIV imeathiri vipi watu wa eneo lako? Kiafya? kiuchumi?
   2. Ni mbinu zipi za kimsingi za kuambukiza HIV katika eneo lako? Kwa maoni yako, ni makundi yepi ambayo yameathirika sana na HIV? Kwa nini unasema hivyo? Ni makundi yapi ambayo yana uwezekano wa kuathirika?
   3. Unapofikiria kuhusu ABCs (kutojamiana, kuwa mwaminifu na kutumia mpira) ili kuzuia maambukizi katika eneo hili, kufuatilia maagizo haya yamefanikiwa kwa kiasi gani? Kwa nini? (Dokeza kwa mbinu ambayo imefanya kazi na ile ambayo haijafanya kazi)
2. **Maarifa na mitazamo kuhusu mbinu**
   1. Ni mbinu ipi ambayo hutumika sana na marika na makundi ya kijamii? Ni nini ambacho inaifanya kujulikana sana?
   2. Umeskia kuhusu madawa ambayo yanaweza kuzuia kupata HIV iwapo yatatumiwa itakikanavyo? Dokeza ili kufahamu tofauti kati ya PEP na PrEP, uliskia nini kuhusu madawa haya? Ulihisi vipi uliposkia kuhusu madawa haya kwa mara ya kwanza?

*Ninataka kukuambia kuhusu mbinu mpya ambayo inazuia watu kutopata HIV. Inaitwa PrEP ya kumezwa. [Soma maelezo ya PrEP ya kumezwa na uyaache eneo wazi mpaka utakapofikia maswali mengine.] PrEP ya kumezwa ni dawa ambayo inamezwa ili kuhakikisha kuwa mtu hapati HIV anapojamiana na mtu ambaye ana virusi.Iwapo uko kwenye hatari ya kuambukizwa, unastahili kumeza dawa siku saba kabla ya hatari na kwa siku 28 mfululizo baada ya kuwa kwenye hatari.Kwa upande mwingine, iwapo upo kwenye hatari ya kuambukizwa kila unapojamiana, unastahili kumeza dawa hii kila siku.*

1. **Maoni yako ni yapi kuhusu mbinu ya PrEP ya kumezwa ya kuzuia maambukizi ya HIV?**
2. **Kukubalika kwa mbinu**
   1. Ni vitu gani ambavyo vinaweza kuwafanya marika wako na makundi ya kijamii wakubali PrEP ya kumeza*?*
   2. Ni makundi yapi unahisi kuwa yatakuwa tayari kukubali matumizi ya PrEP ya kumeza? Dokeza umri, makundi ya kijamii, watu fulani maalum n.k
   3. Ni vitu vipi ambavyo marika wako na makundi ya kijamii wangependa SANA kwa PrEP ya kumeza? Ni vitu vipi ambavyo marika wako hawangependa SANA kwa PrEP ya kumeza?Kutopenda huku kunatosha kuwafanya wao wasimeze dawa?Kwa nini unasema hivyo?
   4. Marika wako watamuonaje mtu ambaye anameza PrEP? Dokeza kwa wasichana waliobaleghe, wanawake wachanga*, FSW, MSM, marika walio na HIV*
   5. Unadhani marika na makundi ya kijamii wanaweza kuhimizana kupata PrEP ya kumeza? Kwa nini? Kwa nini wasihimizane?
3. **Kupata na marika kusaidiana**
   1. Unadhani marika wako watakuwa na uhuru wa kupata PrEP ya kumeza wapi? Kwa nini? Dokeza  *DICES, vituo vya umma na vya kibinafsi, barazas*
   2. Unaweza kwa hiari kuwahamasisha na kuja na marika wako na makundi ya kijamii ili wapate PrEP ya kumeza? Kwa nini? Kwa nini usiweze? Unaweza kuifanya kutoka wapi? Iwapo unafahamu mahali ambapo hii PrEP ya kumeza inapatikana, unaweza kuwaambia ni wapi? Nini kinaweza kukupa motisha ya kufanya kazi vizuri kama mwalimu mwenza wa PrEP?
   3. Iwapo ungekuwa unajua kuwa mwenza wako anashiriki ngono, unaweza kujadili nao kuhusu PrEP ya kumeza? Kwa nini? Kwa nini huwezi? Unaweza kuifanya vipi?
   4. Ni faida gani zinaweza kupatikana iwapo watumizi wa PrEP ya kumeza wangekuwa wanakutana na watumizi wengiine wa PrEP (vilabu vya marika)? Unadhani hii mikutano ipangwe vipi? Mara ngapi na wapi? Ni nini kinaweza kukupa motisha ya kuhudhuria mikutano hii?
   5. Unaweza kubali kuwekwa na pamoja na mtumizi wa PrEP (kama rafiki rika) ili uwape msaada?Kwa nini? Kwa nini huwezi?Unadhani hili linaweza kufanywa vipi?
   6. Unaweza kuzungumza na watu, marafiki na marika kuhusu matumizi yako ya PrEP? Unaweza kuambiana uliyoyapitia? Ni nini unadhani kinaweza kukufanya kufanya hivyo?
   7. PrEP itaathirika kwa kiasi gani na vita, kuogopa kubaguliwa, kuogopa kupoteza wateja? Wenza na biashara? Ni nini kinaweza kusababisha matatizo haya? PrEP inaweza kupeanwa ili kupunguza matatizo haya?
4. **Habari kuhusu PrEP ya kumeza:**
   1. Ni mbinu ipi ya mwafaka ya kuwafikia marika wako na habari kuhusu PrEP ya kumeza? Kwa nini unasema hivyo?Dokeza kwa mitandao ya kijamii, marika, na vyombo vya habari n.k
   2. Ni vitu vipi vya maana ambavyo marika wako wangependa kufahamu kuhusu PrEP ya kumeza?
   3. Ni aina ipi ya ujumbe ambao unaweza kuwafanya marika wako kukubali matumizi ya PrEP ya kumeza?Ni katika hali gani ambamo unaweza kupeana habari kama hii?
   4. Ni habari ipi ya ziada kuhusu PrEP ambayo ungependa kufahamu?

***Peer Educators: Adapted Guide as Scale-Up Progressed: English Guide***

1. **Perceptions about HIV prevention**
   1. What are the current perceptions towards HIV risk among your peers/community members? What explains these observations? Which groups are being perceived to have high risk of new HIV infections?
   2. How is the word, “being at risk’ perceived by your peers/community members? Is the word “being vulnerable” a better choice of words?
   3. What HIV prevention strategies are being utilized among your peers/community members? How has this changed as a result of the introduction of Oral PrEP?
2. **Knowledge and attitudes about KPs**
   1. What is your opinion towards the interaction of your peers/community members and the health system? What has facilitated these interactions? What barriers still exist on the community interaction with KPs? What is facilitating this interaction?
   2. What is your current opinion regarding Oral PrEP as a method of preventing new HIV infections? What explains your description?
3. **Acceptability of PrEP**
   1. What if the current level of acceptability of PrEP as part of the HIV prevention methods among your peers/community members? Why? /Why Not? What things are making your peers/community members accept to use oral PrEP? *What is discouraging your peers*/community members *from using Oral PrEP?*
   2. What are your current views about prescribing PrEP to different populations? *Probe about adolescent girls, young women, FSW, MSM, HIV positive peers*, *transgender*
   3. How much are stigma, discrimination, packaging and violence towards KPs affecting the delivery of PrEP? *How can these be addressed?*
   4. Which access routes are being preferred by your peers/community members to receive PrEP services*? in-reaches, outreaches, moonlights, walk-ins*
4. **Access for PrEP**
   1. Which service delivery points are your peers/community members preferring to access Oral PrEP? Why? Are there differences in preferences between different types of KPs? What explains these differences? *Probe DICEs, private facilities and public facilities*
   2. What barriers do you think your peers/community members encounter when accessing PrEP in the different health facilities? *Waiting times, HIV testing, lack of commodities, many revisits*, *high mobility, monthly refills, lack of privacy, poor attitudes among staff*
   3. What has facilitated the access of PrEP among your peers/community members? *Location of services, good quality services, counselling*
   4. What factors continue to impede your work as a peer educator//community health worker? What has been motivating you to continue mobilizing your peers to access PrEP services? *What can be done to reduce these problems?*
   5. What has been the trends in uptake, adherence, and revisits for PrEP services among your peers/community members? What explains these trends? What can be done improve these indicators? *Probe use of client tracking using peer educators, use of peer clubs, peer buddies, SMS, calling, sending adherence messages, adherence counselling etc*
   6. What suggestions can you make to the health facilities to improve the quality of PrEP services?
   7. How much has the introduction of PrEP affected the use of other prevention methods such as condoms, having few partners, abstinence for the AGYW? *To what extent are your peers reporting more STIs, pregnancies, abortions?*
5. **Information about Oral PrEP:**
   1. What information gaps as far as the mobilization of your peers/community members to receive PrEP is concerned have not been adequately addressed? *Probe for social media, additional training, mentorship, peer-led approaches (on job training), print etc. What can be done to address these gaps?*
   2. What additional information about PrEP would you provide to your peers/community members?

***Peer Educators: Adapted Guide as Scale-Up Progressed: Kiswahili Guide***

1. **Mitazamo kuhusu kuzuia HIV**
   1. Mitazamo ya kisasa kuhusu hatari ya HIV miongoni mwa marika wako na makundi vya kijamii ni ipi?Maelezo ya maono haya ni yepi?Ni makundi yepi ambayo yanachukuliwa kuwa na hatari kubwa ya maambukizi mapya?
   2. Neno, “kuwa katika hatari’ linachukuliwa vipi na marika wako na makundi vya kijamii? “kuwa kwenye mazingira magumu” ni chaguo bora la maneno?
   3. Ni mbinu zipi za kuzuia HIV ambazo zinatumika sana na marika wako na makundi vya kijamii? Haya yamebadilika vipi kama tokeo la kuleta PrEP ya kumeza?
2. **Maarifa na mitazamo kuhusu KPs**
   1. Maoni yako ni yapi kuhusu mtagusano wa marika wako na makundi vya kijamii na mfumo wa afya? Ni nini kimefanikisha mtagusano huu? Ni vizuizi vipi viko kwenye mtagusano kati ya jamii na KPs? Ni nini kinafanikisha mtagusano huu?
   2. Maoni yako ya sasa ni yapi kuhusu PrEP ya kumeza kama mbinu ya kuzuia maambukizi mapya ya HIV? Ni nini kinaeleza maelezo haya?
3. **Kukubalika kwa PrEP**
   1. Kiwango cha sasa cha kukubalika kwa PrEP kama mojawapo ya mbinu ya kuzuia maambukizi ya HIV miongoni mwa marika wako na makundi vya kijamii ni kipi? Kwa nini? Kwa nini sivyo? Ni mambo gani yanafanya marika wako wakubali kutumia PrEP ya kumeza? *Ni nini kinaua marika wako moyo kutotumia PrEP?*
   2. Maoni yako ya sasa kuhusu kuwaandikia matumizi ya PrEP kwenye kikundi fulani cha umma ni yapi? Dokeza kuhusu wasichana waliobaleghe, wanawake wachanga, *FSW, MSM, marika walio na HIV*, *transgender*
   3. Ni kwa kiwango kipi ambacho unyanyapaa, ubaguzi, vipimo na vita vinaathiri upeanaji wa PrEP? Haya yanaweza kuangaziwa vipi?
   4. Ni nyenzo zipi za upatianaji wa huduma za PrEP ambazo zinapendelewa sana na marika wako na makundi vya kijamii? *Semina za ndani, semina za maeneo, moonlights, walki-ins*
4. **Kupata PrEP**
   1. Ni mahali papi pa upeanaji wa huduma za PrEP ya kumeza ambapo marika wako na makundi vya kijamii wanapendelea kupata kutoka? Kwa nini? Kuna tofauti za mapendeleo kati ya aina tofauti ya KPs? Ni nini kinaelezea tofauti hizi? Dokeza *DICEs,vituo vya umma na vya kibinafsi*
   2. Marika wako wanakumbwa na changamoto zipi wakati wa kupata PrEP katika vituo mbalimbali? Muda wa kusubiri? kupima HIV? Ukosefu wa bidhaa, ziara nyingi, kuhama hama, kuendea madawa Zaidi, ukosefu wa ufaragha*, uroho mbaya miongoni mwa wahudumu*
   3. Ni nini kimefanikisha kupata kwa PrEP miongoni mwa marika na makundi vya kijamii? Mahali pa huduma, ubora wa huduma, mashauriano
   4. Ni nini kinazuia kazi yako kama mwalimu rika? Ni nini kimekuwa kikikumotisha kuendelea kuwahamasisha marika wako kuenda kupata huduma za PrEP? *Ni nini kinaweza kufanywa ili kupunguza matatizo haya?*
   5. Mtindo wa kumeza, kuzingatia na kurudia huduma za PrEP miongoni mwa marika na makundi vya kijamii? Ni nini kinaelezea mtindo huu? Ni nini kinaweza kufanywa ili kuboresha mtindo huu? Dokeza kwa kufuatilia kwa mteja kwa ujumbe mfupi, kupiga simu, kutuma ujumbe wa mazingatio, kufuatilia mashauri ,kutumia mwalimu rika, kutumia vilabu vya marika, marafiki marika, *n.k*
   6. Ni mapendekezo gani utawapa vituo vya Afya ili kuboresha huduma za PrEP?
   7. Kuleta PrEP kumeathiri vipi matumizi ya mbinu zingine za kuzuia maambukizi mfano mipira, kuwa na wenza wachache, kutojamiana kwa AGYW? Ni kwa kiwango kipi wenza wako wanaripoti magonjwa ya zinaa, mimba na kuavya mimba?
5. **Habari kuhusu PrEP ya kumeza:**
   1. Kuna mianya ipi ihusianayo na kuhamasisha marika wako na makundi vya kijamii kuchukua PrEP ambayo haijaangaziwa kwa kina?Dokeza kwa mitandao ya kijamii, mafunzo zaidi, walimu marika, kufunzwa kazini *, magazeti n.k. Ni nini kinaweza kufanywa kuangaziwa mianya hii?*
   2. Ni habari ipi ya ziada kuhusu PrEP ambayo ungependa kufahamu?

**Appendix C: Health Manager KII Guide**

***Health Managers: Formative Project Phase (early 2018): English Guide***

1. **HIV burden and prevention**
   1. To what extend is HIV a significant public health problem in the health system? How has it affected the country? *Health? Economically?* *Socially?*
   2. What are the current HIV transmission patterns? How is this affected by adoption of HIV prevention approaches? What milestones have been achieved by recent changes in HIV prevention?
   3. What is your opinion about the key groups currently driving new HIV infections in Kenya?
2. **Knowledge and attitudes about methods**
   1. What barriers have been impeding HIV prevention in Kenya? What has been facilitating HIV prevention efforts in Kenya?
   2. What Biomedical interventions are currently being offered for HIV prevention in your area? *Probe to differentiate PEP and PrEP*. Have you heard about PrEP?

*I want to tell you about a new measure that is being introduced to prevent people from contracting HIV. It is called Oral PrEP. Read the Oral PrEP description and keep it displayed until you come to the next set of questions. Oral PrEP is a drug that is taken to ensure that one does not contract HIV when having sexual intercourse with an infected person. If you are at risk of being exposed, you are required to take the drug seven days prior to exposure and for 28 days after possible exposure. On the other hand, if there is a possibility of you being exposed at every sexual encounter you are required to take the drug on a daily basis.*

1. **What is your opinion regarding Oral PrEP as a method of preventing new HIV infections? Why?**
2. **Priority of PrEP as an intervention approach**
   1. Is Oral PrEP something the health system can advocate as an additional method for HIV prevention? Why? /Why Not? *Probe moral, social, health, access, economic reasons, human rights, public health reasons*
   2. Which groups would benefit most from receiving Oral PrEP? *Probe age, key populations, social groups etc.* Why?
   3. What are the key components the health system should be put in place before adoption PrEP as an intervention approach? *Commodities, personnel, training, supervision, mentorship, tools etc*
   4. What issues should health managers address to enhance adoption of PrEP by those who can most benefit from it? *Workload, infrastructure, fit as an integrated model, identifying access points, attitudes towards users, commodity management*
   5. How would health providers perceive those who are taking Oral PrEP? *Probe about adolescent girls, young women, FSW, MSM, transgender, HIV positive peers, Probe promiscuity, immoral, reckless etc.* Why?
   6. What are the health systems priorities towards delivery of prevention services to *FSW? And MSM/MSWs*? How is the health system designed to facilitate access and use of PrEP by these groups*? How about sexually active adolescent girls and young women?*
3. **Access of PrEP services**
   1. Where do you think users of PrEP would be most comfortable accessing Oral PrEP? Why?
   2. How can users be mobilized to receive Oral PrEP? Why? Why Not? What can motivate the mobilization of users to access PrEP services? *Peer educators,* *health talks, launches, training*
   3. To what extend can the use of PrEP be affected by stigma and discrimination towards the users, existing laws? Why? *What can be done to address these challenges so that PrEP can benefit the community?*
   4. What, according to your opinion is needed for a health facility to deliver PrEP? How would the community react if the health facility they seek care services from is offering PrEP? *Probe and if KPs are attending the same facility*
4. **Information about Oral PrEP:**
   1. Which methods would be most suitable to reach potential users with information about oral PrEP? Why? *Probe for social media, peer-led approaches, print, mass media, baraza, edutainment etc.*
   2. What information about Oral PrEP do you think would be most relevant to your community?
   3. Under what circumstances would health managers provide this information? And how can that be facilitated?

***Health Managers: Formative Project Phase (early 2018): Kiswahili Guide***

1. **Ufahamu na mitazamo kuhusu kuzuia HIV**
   1. HIV ni tatizo kwa kiasi gani katika eneo lako?imeathiri vipi watu katika eneo lako?Kiafya?kiuchumi?kijamii?
   2. HIV hupitishwa kwa njia gani kwenye eneo lako?
   3. Kwa maoni yako, ni marika wa umri upi ambao kwa kiasi kikubwa WANAWEZA KUWA WAMEAMBUKIZWA HIV katika eneo lako? Ni marika wa umri upi ambao wameathirika sana na HIV? Kwa sababu gani?
   4. Ni mbinu zipi za kuzuia HIV zinazopatikana katika eneo lako?Dokeza kuhusu *ABCs (kutojamiana, kuwa mwaminifu na kutumia mpira)* ABCs imefanikiwa kwa kiasi gani? Kwa nini? Kwani nini haijafanikiwa? (dokeza kwa vipimo ambavyo vinafanya kazi na vile havifanyi kazi )
2. **Mitazamo na matendo ya mbinu ya kuzuia HIV.**
   1. Ni mbinu zipi ambazo makundi ya umri tofauti hutumia kwa sana? Ni nini unadhani kinaweza kuelezea mapendeleo haya?
   2. Umeskia kuhusu madawa ambayo yanaweza kuzuia kupata HIV iwapo yatatumiwa itakikanavyo? Dokeza ili kufahamu tofauti kati ya PEP na PrEP, uliskia nini kuhusu madawa haya? Ulihisi vipi uliposkia kuhusu madawa haya kwa mara ya kwanza?

*Ninataka kukuambia kuhusu mbinu mpya ambayo inazuia watu kutopata HIV. Inaitwa PrEP ya kumezwa. [Soma maelezo ya PrEP ya kumezwa na uyaache eneo wazi mpaka utakapofikia maswali mengine] PrEP ya kumezwa ni dawa ambayo inamezwa ili kuhakikisha kuwa mtu hapati HIV anapojamiana na mtu ambaye ana virusi.Iwapo uko kwenye hatari ya kuambukizwa, unastahili kumeza dawa siku saba kabla ya hatari na kwa siku 28 mfululizo baada ya kuwa kwenye hatari.Kwa upande mwingine, iwapo upo kwenye hatari ya kuambukizwa kila unapojamiana, unastahili kumeza dawa hii kila siku.*

1. **Maoni yako ni yapi kuhusu mbinu ya PrEP ya kumezwa ya kuzuia maambukizi ya HIV? Maoni yako ni yapi kuhusu mbinu ya PrEP ya kumezwa ya kuzuia maambukizi ya HIV katika eneo lako? Kwa nini?**
2. **Kukubalika kwa PrEP**
   1. PrEP ya kumeza ni mbinu ambayo watu wa eneo lako wangependekeza iongezwe kama mbinu zaidi ya kuzuia maambukizi ya HIV? Kwa nini? Kwani nini isiweze? Dokeza Maadili, kijamii, kiafya, upatikanaji, sababu za kiuchumi, haki za kibinadamu, sababu za Afya ya umma
   2. Ni makundi yapi kutoka eneo lako unadhani yatafaidi sana kwa kutumia PrEP ya kumeza? Dokeza miaka, idadi ya watu maalum, makundi fulani ya kijamii n.k Kwa nini?
   3. *Watu wa kutoka eneo lako watamwonaje mtu ambaye anameza PrEP?* Dokeza kuhusu wasichana waliobaleghe, wanawake wachanga*, FSW, MSM,marika walio na virusi, Dokeza kuwa na wenza wengi, bila maadili, asiyejali n.k.* Kwa nini? Kwani nini asiweze?
   4. Mtazamo wa eneo lako kwa *FSW ni upi? na MSM/MSWs*? Hii inaathiri vipi upatikanaji na matumizi ya PrEP*? Na vipi kuhusu wasichana waliobaleghe na wanawake wachanga ambao wanashiriki ngono?*
3. **Upatikanaji wa huduma ya PrEP**
   1. Unadhani wanaoweza kutumia katika eneo lako watakuwa huru kupata wapi PrEP ya kumeza?Kwa nini?
   2. Ni njia zipi zinazoweza kuwaleta pamoja ili wapokee PrEP ya kumeza wanaoweza kutumia? Kwa nini? Kwani nini wasiweze?Iwapo unafahamu mahali unaweza kuipata hii PrEP ya kumeza, unaweza kuwahamasisha watumizi ili waipokee hiyo PrEP ya kumeza?Kwa nini? Kwani nini usiweze?Nani anaweza kukuhimiza kufanya hivyo?
   3. Unaweza taka kuzungumza na watu, marafiki na mnaofanya nao kazi kuhusu PrEP ya kumeza? Kwa nini? Kwani nini usiweze?
   4. Matumizi ya PrEP yanaweza kuathirika vipi na unyanyapaa na kubaguliwa kwa watumizi, sheria zilizoko?Kwa nini? Ni nini kinaweza kufanywa kuangazia changamoto hizi ili PrEP iweze kufaidi watu wanaoishi katika eneo lako?
   5. Ni nini, kulingana na maoni yako, kinahitajika kwenye kituo cha Afya ili kupeana PrEP?Watu wa eneo lako watafanya nini watakapogundua kuwa kituo ambamo wanapata huduma kutoka kinapeana PrEP?Dokeza iwapo KPs wanahudhuria kituo hicho
4. **Habari kuhusu PrEP ya kumeza:**
   1. Ni mbinu zipi mwafaka ambazo zinaweza kutumika kuwafikia watu wanaoishi katika eneo lako na habari kuhusu PrEP ya kumeza?Kwa nini?Dokeza kwa mitandao ya kijamii, marika kutumika, vyombo vya habari, baraza na maburudisho ya kuelimisha n.k
   2. Ni habari zipi kuhusu PrEP ya kumeza ambazo unadhani zinaweza kuwahusu watu wanaoishi katika eneo lako?
   3. Ni mambo yepi yanayoweza kupelekea viongozi wa eneo lako kupeana habari?Haya yanaweza kufanyika vipi?

***Health Managers: Adapted Guide as Scale-Up Progressed: English Guide***

1. **HIV prevention**
   1. What are the current perceptions towards the adoption of PrEP as part of the national HIV prevention package? *What explains this trend?*
   2. To what extent has provision of PrEP been integrated as a routine health service? *What issues are impeding integration? What facilitating factors have improved integration of PrEP in private facilities, public facilities and drop in centres?*
2. **Attitudes towards KPs**
   1. How is the provision of services to KPs being perceived by the health system? *What has facilitated these perceptions? What barriers still need to be addressed to change perceptions about the provision of services for KPs?*
   2. What is your current opinion regarding scaling-up Oral PrEP as a complimentary method for preventing new HIV infections among KPs? *What explains your description?*
3. **Acceptability for institutions to support PrEP**
   1. What is the current level of acceptability of PrEP as part of the HIV prevention methods by financing and implementing institutions? *Why? /Why Not?* What factors are preventing institutions from supporting oral PrEP? *Probe stigma, discrimination, religious conflicts, resource constraints, priorities, existing laws, human rights issues*
   2. To what extent are County and the National Governments willing to allocate resources to support implementation of the PrEP scale-up? *What needs to be done to mobilize governments to allocate resources?*
4. **Service delivery for PrEP**
   1. What service delivery channels are reporting higher numbers of eligible clients accessing Oral PrEP? What explains differences in preferences between different types of KPs? *Probe DICEs, private facilities and public facilities*
   2. What health system barriers are preventing uptake of PrEP? *Waiting times, lack of commodities, many revisits*, *high mobility, monthly refills, lack of privacy, poor attitudes among staff. How can these barriers be addressed?*
   3. What health system factors are facilitating the delivery of PrEP? *Training, commodities, supervision, mentorship, evidence informed decision making, media coverage, resource allocation, it is the new thing, monitoring and evaluation, knowledge flow*
   4. What suggestions can you make to HIV implementing partners to improve the uptake and quality of PrEP services?
   5. How much has the introduction of PrEP affected implementation of other prevention methods? How has this affected the national health priorities?
   6. From a managerial point of view, how does the future for PrEP look like? *Budget allocation, scale up, high priority within the donor community*
5. **Information about Oral PrEP:**
   1. What information gaps regarding the implementation of PrEP is missing among senior managers? *What can be done to address these gaps?*
   2. What appropriate steps can be adopted to mobilize collaboration between partners to encourage willingness to participate in the scale up?

***Health Managers: Adapted Guide as Scale-Up Progressed: Kiswahili Guide***

1. **Ufahamu na mitazamo kuhusu kuzuia HIV**
   1. Mitazamo ya sasa kuhusu hatari ya HIV katika eneo lako ni ipi? Maelezo ya maono haya ni yepi? Ni watu gani wanaoishi katika eneo lako ambalo wanachukuliwa kuwa kwenye hatari zaidi ya maambukizi mapya?
   2. Ni mbinu zipi za kuzuia HIV ambazo zinajulikana sana katika eneo lenu? Dokeza  *ABC, PEP, PrEP, TasP.* Hizi zimebadilika vipi tangu kuletwa kwa PrEP ya kumeza?
2. **Ufahamu na mitazamo kuhusu KPs**
   1. Maoni ya sasa hivi ni yepi kuhusu watu maalum(FSW, MSM/MSW, transgender) katika eneo lako?Ni nini kinaelezea mitazamo hii*? Mitazamo hii inabadiliisha vipi namna eneo linatagusana na watu hawa? Ni mapendekezo gani unaweza kufanya ili kuboresha mitazamo ya maeneo haya kwa KPs?*
   2. Maoni yako ya sasa kuhusu PrEP ya kumeza kama mbinu ya kuzuia maambukizi mapya ya HIV katika eneo lako ni yepi? Je, na kuhusu upeanaji wa PrEP kwa KPs, AGYW?
3. **Kukubalika PrEP**
   1. Kiwango cha kukubali PrEP ya kumeza kama mbinu ya kuzuia maambukizi katika eneo lako ni kipi? Kwa nini? Ni mambo yepi yanawafanya watu katika eneo lako kukubali matumizi ya PrEP ya kumeza *Ni nini kinaua moyo watu kutoka eneo lako kutotumia PrEP ya kumeza?*
   2. Maoni yako ni yapi kuhusu kumwandikia msichana aliyebaleghe , mwanamke mchanga na wenza ambao hawana HIV na wapo kwenye mahusiano ya mmoja kuwa na HIV na mwingine hana?
   3. Ni kwa kiwango kipi unyanyapaa, ubaguzi, mitazamo ya kidini na vita kwa FSW, MSM/MSM, AGYW wanaojamiana yanaathiri upeanaji wa PrEP ya kumeza?*Haya yanaweza kuangaziwa vipi?*
   4. Ni njia zipi za kupata PrEP ambazo zinapendelewa na watu wanaoishi katika eneo lako kupokea huduma za PrEP? Mafunzo ya ndani, mafunzo ya maeneo  *moonlights, walk-ins,kuongozwa kwa marika*
4. **Kupata for PrEP**
   1. Ni njia zipi ambazo watu wa eneo lako wanapenda kuzitumia kupata PrEP ya kumeza?Kwa nini? Ni nini kinaelezea utofauti katika watumizi tofauti? *Dokeza DICEs, Vituo vya kibinafsi na vituo vya umma*
   2. Ni vizuizi vipi ambavyo vinawakumba watumizi wa PrEP kutoka eneo lako wakati wa kupata PrEP katika vituo mbalimbali vya Afya? *Muda wa kusubiri, kupimwa HIV, kukosa vifaa, mara nyingi ya kuzuru,kuhama hama, kuchukua dawa kila mwezi, hakuna ufaragha, hisia hasi kutoka kwa wahudumu*
   3. Ni mambo gani yanakuza matumizi ya PrEP katika vituo? Dokeza mteja, mfumo wa kiafya, wasimamizi wenyeji
   4. Ni mapendekezo gani unaweza kuyafanya kwa nyenzo mbalimbali za upeanaji huduma katika eneo lako ili kuboresha huduma ya PrEP ya kumeza?
   5. Kwa maoni yako mwenyewe, kuletwa kwa PrEP ya kumeza kumeathiri vipi matumizi ya mbinu zingine za kuzuia maambukizi mfano kutojamiana? Mipira?mwenza mmoja mwaminifu?Ni kwa kiwango kipi magonjwa ya zinaa, mimba na kuavya mimba yanaripotiiwa katika eneo lako?
5. **Habari kuhusu PrEP ya kumeza:**
6. Ni mianya ipi kuhusu PrEP ya kumeza ambayo haijaangaziwa vizuri katika eneo lako?Ni makundi yapi hayajafikiwa na habari ya kutosha?Ni nini kinaweza kufanywa ili kuangazia mianya hii?Ni nyenzo zipi zinazoweza kutumika kupeana habari hii kwa wale ambao wanahitaji sana?
7. Ni habari ipi ya ziada ungependekezea watu wanaoishi katika eneo?

**Appendix D: Data Repository**

Data are available at this link: <https://clinepidb.org/ce/app/workspace/analyses/DS_d70aacce42/new/details>
